# Perceived value but persistent barriers: A qualitative study of healthcare worker experiences with the Impilo electronic health record system in rural Zimbabwe

**DOI:** 10.1371/journal.pdig.0001765

**Published:** 2026-10-08

**Authors:** Shalom Rudorwashe Dube, Perez Livias Moyo, Methembe Yotamu Khozah

**Affiliations:** Department of Environmental Health, Faculty of Environmental Science, National University of Science and Technology, Bulawayo, Zimbabwe; North Carolina A&T State University: North Carolina Agricultural and Technical State University, UNITED STATES OF AMERICA

## Abstract

Electronic Health Records (EHRs) are pivotal for improving healthcare data quality and service delivery. Despite the introduction of the Impilo EHR system at St Anne’s Mission Hospital in Brunapeg, utilisation remains low. This study aimed to explore healthcare workers’ (HCWs) experiences and identify the key barriers and facilitators to Impilo’s utilisation. A descriptive qualitative study was conducted at St Anne’s Mission Hospital. In-depth interviews were conducted with 20 HCWs authorised to use Impilo, selected via purposive sampling until thematic saturation was achieved. Thematic analysis, following the Braun and Clarke approach, was used to analyse the data. Trustworthiness was ensured through multi-coder analysis, the maintenance of an audit trail, and thick descriptive reporting. HCWs reported positive experiences related to ease of data access, improved communication, and better inventory management. However, significant barriers undermine utilisation. The analysis revealed three key themes: (1) Benefits of Impilo, (2) Multifaceted Challenges, and (3) Suggested Improvements. Challenges included pervasive technical and infrastructural issues (e.g., frequent network downtime, lack of offline functionality), human resource constraints leading to dual documentation and increased workload, and subsequent compromised data quality due to inconsistent entry. The underutilisation of the Impilo system is not due to a lack of perceived value but is primarily driven by remediable barriers related to infrastructure, training, and workflow design issues. For successful implementation, interventions must be multifaceted, addressing technical reliability (e.g., offline functionality), human capacity (continuous training and support), and organisational processes (dedicated data clerks and inclusive system redesign). This study provides a framework for improving EHR adoption in similar low-resource rural settings.

## Author summary

In this study, we spoke with healthcare workers at a rural hospital in Zimbabwe to understand their experiences using a new digital health record system called Impilo. We discovered that while staff see great value in the system, it makes finding patient information faster, reduces errors, and helps manage medical supplies; its potential is largely untapped. The primary reasons for this are not a rejection of the technology, but rather solvable, real-world problems. Frequent internet outages, a lack of training, and the exhausting burden of having to record the same information on both paper and the digital system are the main hurdles. The findings suggest that for digital health tools to succeed in rural and low-resource settings, the focus must extend beyond the software itself. Success depends on creating a supportive environment, which includes reliable infrastructure like offline functionality, ongoing staff training and support, and a fundamental redesign of workflows to reduce the double burden on healthcare workers. We believe that by addressing these practical barriers, our work can help guide the successful adoption of digital health systems not only in Zimbabwe but in similar communities worldwide, ultimately strengthening healthcare delivery for all.

## Introduction

Quality healthcare data are key to decision-making and improving service delivery. Data quality is ensured throughout the process before collection, during collection, analysis, storage, and accessibility [1]. The characteristics of usable data include “availability, accessibility, accuracy, validation, currency, completeness, consistency, usability, provenance, security, and confidentiality [2]. Policymakers, researchers, program managers, donors, non-governmental organisations (NGOs), the public, and other stakeholders have a growing demand for health-related data. Consequently, there is a need for a comprehensive and clearly defined data collection and reporting system. This system should encompass health facilities at the local level and extend to the national level, catering to the needs of all data users [3].

Since the 20^th^ century, primary healthcare documentation and data collection have been conducted using paper records. The collection of healthcare data has changed significantly over the years. Advancements in technology have triggered a shift, changes in healthcare procedures, and a growing demand for accurate and comprehensive health information [4]. EHRs serve as a tool for collecting healthcare data by providing a comprehensive picture of a patient’s care and managing health information digitally. EHRs provide a comprehensive picture of a patient’s care and can create and manage health information digitally [5]. Hence, EHRs are crucial sources of information for various health systems. These systems are used by public health officials, clinicians treating individual patients, and researchers studying population health, as well as the determinants of health and treatment efficacy [6].

When implemented correctly, EHR systems can improve the standard of care provided to patients. This can be achieved through increased compliance with guidelines and a reduction in medication errors and adverse drug effects [7]. The benefits of instant data retrieval and improved disease surveillance through timely access to outbreak reports have been reported by other scholars [5,8]. Moreover, the use of standardised data with key indicators allows for easier access to all of the patient’s medical histories, reducing the time spent searching for previous laboratory results and recalling medical history [7,8]. This also helps prevent unnecessary repetition of tests previously conducted and reduces errors resulting from illegible handwriting [8]. Additionally, the use of electronic health records improves continuity of care among different healthcare providers and provides useful reminders [8].

The United States and the United Kingdom are considered pioneers in the adoption and implementation of EHRs [9]. In the United States, the introduction of the American Recovery and Reinvestment Act in 2009 saw an increase in the adoption and utilisation of EHRs by more than two-fold. Adoption was lower in facilities that were not eligible for federal meaningful use incentives [10]. In a European Region report by the World Health Organization (2016), 56% of member states had a national EHR system. Utilisation across primary, secondary, and tertiary facilities was 64%, 63%, and 55%, respectively. There was also a complementary use of systems such as laboratory information and pharmacy information systems. European countries have also reported issues of funding as barriers to EHR implementation, and in secondary care, progress has been slow due to differences in regional and local systems’ data practices [11].

African countries generally still lag in the availability and utilisation of EHRs, especially in primary health care facilities [12–14]. African countries are characterised by the widespread use of open-source EHRs such as DHIS, Epi Info, Open MRS, and Open EMR [12]. Specialised ones are more administrative than clinical [13]. While some have been implemented successfully, it has been through international support and biased programs such as HIV/AIDS, leaving a question of long-term feasibility [15]. In Ethiopia, recent studies have highlighted persistent barriers to accurate data documentation during institutional births, with HCWs citing workload and infrastructure limitations as key challenges [16]. Similarly, research applying the UTAUT3 model in Ethiopia found that facilitating conditions and social influence are critical predictors of HCWs’ intention to use EHR systems [17]. In Nigeria, implementation has been observed across states, but with poor consistency in utilisation [15]. In Tanzania, despite widespread adoption, the country still lacks effective utilisation of EHRs [18]. Zimbabwe’s public hospitals partially adopted EHRs and used them in specific departments and for HIV/AIDS management. Despite the adoption of EHRs, the study found that most data is still stored in paper-based systems [14].

As part of the national EHR integration process, the Ministry of Health and Child Care (MoHCC) aims to decommission as many Paper-Based Registers (PBR) as possible, assisting with patient care and management, tracking patients, and generating current reports and registers using the EHR [19]. Since February 2015, the Ministry of Health and Child Care (MoHCC) of the Government of Zimbabwe (GoZ) has been involved in the design, development, and testing of a new electronic health record (EHR) system called “Impilo,” [19]. As of July 2023, 1,023 out of 1800 health facilities had implemented Impilo.

At St Anne’s, Impilo was implemented in the Fourth quarter of 2022. Based on departmental needs, electronic tablets were distributed to facilitate data collection. HCWs are the end users of Impilo. Their performance ultimately influences the utilisation and effectiveness of the system. However, there is a notable gap in qualitative research exploring the lived experiences of HCWs using EHRs in Zimbabwean settings. While quantitative studies have documented low utilisation rates [20,21]. The “why” behind these statistics remains underexplored. Qualitative methods are essential for capturing the contextual, emotional, and practical nuances that shape HCWs’ daily interactions with digital health tools. An insight into their experiences, challenges faced and suggested areas of improvement can help tailor Impilo to meet the needs of HCWs, leading to improved utilisation and ultimately service delivery. Quality healthcare data is crucial because it guides decision-making and healthcare service delivery. Completeness, availability, and accessibility are some key components of quality data [1]. Reliable data collection tools can improve data quality.

St Anne’s continues to use Paper-Based Records/Registers (PBRs), which have their own set of challenges that compromise data quality. In a hospital survey done at St Anne’s, challenges with PBRs included the destruction of PBRs by termites, torn pages from the registers by daily staff handling, and an overcrowded archive that lacks an orderly manner of organising records for retrieval. These challenges make data from PBRs incomplete, unavailable and inaccessible. Timely retrieval of PBRs in an emergency proves difficult. A missing test result may lead to delays in an emergency theatre case. In 2022, a transition aimed at producing improved data quality saw the introduction of Impilo at St Anne’s Mission Hospital. Unlike PBRs, Impilo offers online data storage and backup, making it resilient to destruction. They can store large amounts of data, with easy retrieval [19].

However, HCWs still predominantly use PBRs at St Anne’s Mission Hospital. In a support and supervision register on 03 September 2023, it was highlighted that utilisation of Impilo was generally low. The district team highlighted the need to seek assistance from the health information department and encouraged improvement in attitude towards the utilisation of Impilo. On 04 September 2024, the district team documented that performance on Impilo was below 50%, with the ANC booking indicator the only one with 100% data entry. Despite initial training interventions conducted, including the initial training of three HCWs in 2022 and a five-day training of 10 HCWs in 2023, and the District team visits, low utilisation persists.

This study seeks to qualitatively explore the experiences of Health Care Workers who use Impilo at St Anne’s, identify barriers to full utilisation of Impilo and factors which might be adopted to improve utilisation. This study might contribute evidence-based recommendations that may inform the Ministry of Health and Child Care, the Impilo technical team and relevant management to recognise and address these barriers to improve Impilo utilisation and contribute to improved health service delivery.

## Materials and methods

### Study design

A descriptive qualitative study design was employed. This design was selected to provide a rich, detailed, and contextual understanding of healthcare worker’s experiences, perceptions, and suggestions regarding the Impilo EHR system. From an epistemological perspective, this study adopts an interpretive/constructivist stance, recognising that HCWs’ experiences are shaped by their unique social, professional, and environmental contexts. Qualitative methods are particularly suited to this inquiry because they allow for the exploration of lived experiences, capture unanticipated phenomena, and provide the depth necessary to understand the “why” behind observed behaviours such as low system utilisation [22,23].

### Study setting

The study was conducted at St Anne’s Mission Hospital - Brunapeg, a rural hospital in the Mangwe District of Matabeleland South Province, Zimbabwe. It serves a catchment population of approximately 3,073 people annually and is one of 13 health facilities in the district [24].

### Study population and sampling

The target population was all 43 HCWs at St Anne’s Mission Hospital authorised to enter data into the Impilo system across various departments (Outpatient Department, Maternity, Laboratory, Pharmacy, etc.). A purposive sampling technique was employed to ensure a diversity of roles, departments, and experience levels. The sampling process involved identifying potential participants from departmental lists, then selecting individuals who represented different professional categories (nurses, doctors, pharmacists, nurse aides, environmental health technicians, and laboratory technicians) to capture a wide range of perspectives. Recruitment continued until thematic saturation was achieved, defined as the point at which no new themes or insights emerged from subsequent interviews [25], which occurred after including 20 participants.

### Data collection tools and procedure

A semi-structured interview guide was used to conduct face-to-face, in-depth interviews. The guide (see Table 1) was designed based on the study objectives, which focused on system usability, data quality, workflow changes, and suggestions for improvement, and was informed by a literature review of EHR implementation challenges in low-resource settings. The guide was piloted with two HCWs (not included in the study) to check for clarity and flow, leading to minor phrasing adjustments. Interviews were conducted in a private consultation room at St Anne’s Mission Hospital to ensure confidentiality and minimise interruptions. Before each interview, the researcher explained the study’s purpose, assured confidentiality, answered all questions, and obtained written informed consent to build rapport and trust. The average interview duration was 22 minutes (range: 15–35 minutes). With participant permission, interviews were audio-recorded. For the two participants who declined recording, detailed verbatim notes were taken. All audio recordings were transcribed verbatim. Field notes were also maintained to capture non-verbal cues and contextual observations.

**Table 1:** Semi-Structured Interview Guide.

Domain	Sample Questions
System Usability	Can you describe your overall experience using Impilo? What do you find easy or difficult about using Impilo on a typical day?
Data Quality	How does Impilo compare to paper-based records in terms of data completeness and accuracy? Can you tell me about a time when you could or could not trust the data in Impilo?
Workflow Changes	How has Impilo changed the way you do your daily work? Can you describe the process of recording a new patient from start to finish?
Barriers	What are the biggest challenges you face when trying to use Impilo? Can you tell me about a time when you wanted to use Impilo but could not?
Facilitators & Improvements	What would make it easier for you to use Impilo regularly? If you could change one thing about Impilo, what would it be?

### Data analysis

Thematic analysis, following the six-phase approach of Braun and Clarke (2019), was used to analyse the data [26]. The process was as follows: (1) familiarisation with the data through repeated reading of transcripts and listening to audio recordings; (2) generation of initial codes using a line-by-line approach; (3) searching for themes by collating codes into potential themes; (4) reviewing themes against the coded data and entire dataset; (5) defining and naming themes; and (6) producing the final report. A multi-coder approach was used: three authors (SRD, PLM and MYK) independently coded the first five transcripts and then met to compare codes. Discrepancies in coding were resolved through consensus meetings. The remaining transcripts were coded by SRD, with regular debriefing sessions with PLM and MYK to ensure consistency. Codes were iteratively refined throughout the analysis process, and themes were developed through team-based discussion and refinement.

### Trustworthiness

Trustworthiness was ensured through several strategies. Credibility was enhanced through multi-coder analysis, peer debriefing sessions, and member checking (preliminary findings were presented to five participants who confirmed they accurately reflected their experiences). Dependability was ensured through the maintenance of an audit trail documenting all analytical decisions, from raw data to final themes. Confirmability was achieved by grounding all themes in participant quotations and maintaining reflexivity notes in which the researchers documented their assumptions and potential biases. Transferability was supported through thick descriptive reporting, providing sufficient contextual detail about the study setting, participants, and implementation environment to allow readers to assess applicability to other contexts.

### Ethical considerations

Ethical approval was obtained from the National University of Science and Technology Institutional Review Board (Ref: NUST/IRB/2025/39). Permission was also obtained from the Ministry of Health and Child Care and the hospital administration at St Anne’s Mission Hospital. All participants provided written informed consent before participation. To protect privacy, interviews were conducted in a private room, no identifying information was recorded, participant codes (KI_1 to KI_20) were used instead of names, and all data were stored on password-protected devices with access limited to the research team. Participants were informed that they could withdraw at any time without consequences.

## Results

### Demographic characteristics of participants

The 20 interview participants represented a range of professional roles, including nurses, doctors, pharmacists, nurse aides, and environmental health professionals, ensuring diverse perspectives. The sample consisted of 12 females (60%) and 8 males (40%), with ages ranging from 25 to 50 years. Years of experience using Impilo ranged from 1 to 2 years, reflecting that the system had been implemented in late 2022. See Table 2 for detailed demographics.

**Table 2:** Interview Respondents’ Demographics (n = 20).

Code	Sex	Age	Role	Years Using Impilo
KI_1	Female	25	Doctor	1
KI_2	Female	26	Nurse	2
KI_3	Male	38	Nurse	2
KI_4	Male	33	Pharm-tech	2
KI_5	Female	45	Nurse Aide	1
KI_6	Male	41	EHT	2
KI_7	Female	45	Nurse Aide	1
KI_8	Female	30	Nurse	2
KI_9	Female	40	Nurse	2
KI_10	Male	31	Doctor	1
KI_11	Female	29	Nurse	1
KI_12	Male	35	Doctor	2
KI_13	Female	42	Nurse Aide	1
KI_14	Female	28	Nurse	2
KI_15	Male	50	Pharm-tech	2
KI_16	Female	26	Nurse	1
KI_17	Male	39	EHT	2
KI_18	Female	44	Nurse Aide	1
KI_19	Female	32	Nurse	2
KI_20	Male	36	Lab Technician	2

EHT = Environmental Health Technician; Pharm-tech = Pharmacy Technician

### HCW experiences with Impilo

Thematic analysis of the interviews revealed three main themes: Benefits, Challenges, and Suggested Improvements, each with several sub-themes (Table 3).

**Table 3:** Themes and subthemes from qualitative interviews.

Theme	Sub-themes
Benefits of using Impilo	Efficiency and data accessibility Clear and effective communication Enhanced inventory and resource management
Challenges of using Impilo	Compromised data quality and integrity Technical and infrastructural barriers Human resources and workflow strain System design and inclusivity gaps
Suggestions for improvement	Human resource and workflow optimisation Technical and infrastructural enhancements Participatory system redesign

### Benefits of using Impilo at St Anne’s Mission Hospital

#### Efficiency and data accessibility.

Participants consistently highlighted that Impilo enabled easy and rapid data retrieval, a significant improvement over paper-based records. They noted that PBRs were easily misplaced, damaged, or not always immediately accessible to all staff members. With Impilo, HCWs could access patient information from any department without being physically present. Additionally, participants valued the system’s automated prompts and reminders, which helped ensure that all relevant information fields were completed a feature that reduced omissions commonly seen with PBRs.

*“I do not need to go to a department to get notes on a patient in case they are missing”.* KI_2

*“I easily log in and retrieve the information I need without having to peruse through a number of pages from the register”.* KI_1

“*Though it is annoying at times, Impilo will not allow me to move to the next stage without filling in key information. This is important because we deliver full information which is usable by anyone else”.* K1_3

#### Clear and effective communication.

Another important benefit identified by participants was the system’s ability to facilitate clear communication across departments without the need for physical presence or handwritten notes. HCWs reported that Impilo eliminated medication errors commonly associated with the misinterpretation of illegible handwriting on paper prescriptions. The ability to access patient information including vital observations and previous orders from different locations streamlined clinical decision-making and reduced redundant questioning of patients and colleagues.

“*I can make a prescription whilst in a different department, and it is carried out in the ward. I can retrieve the patient’s information, like vital observations and previous orders, by the click of a button”.* KI_10

*When I use Impilo, I do not have to ask twice for information about a patient. Some handwritten documents are hard to read, you have to ask the person to tell you what they wrote, sometimes you have to guess”.* KI_12

#### Inventory and resource management.

The pharmacy department and ward staff highlighted that Impilo significantly improved their ability to track stock and manage medical supplies. The system’s automated prompts alerted staff to medicines nearing expiry, which was difficult to track on paper. In the wards, Impilo helped staff keep track of ward stocks and improved accountability by directly linking dispensed items to the logged-in user a feature that paper-based systems could not provide.

*“Tracking expiry dates on paper is difficult and gets overwhelming. We easily miss some medicines nearing expiry only to notice on dispensing. Impilo will just send a pop-up reminder”.* KI_4

*“Once someone dispenses medicine via Impilo, it is directly accredited to the user logged in at the time, unlike on paper, where someone can just retrieve stock without balancing out stock cards”.* KI_15

### Challenges faced when using Impilo at St Anne’s Mission Hospital

#### Data quality and integrity.

Despite the acknowledged benefits of instant data retrieval and automated prompts, participants reported significant concerns about data quality and integrity. The failure to consistently enter data across all departments meant that the information on Impilo was often incomplete, making the system less useful for clinical decision-making. HCWs described a tedious data verification process, with few staff members bothering to verify Impilo data, leaving its integrity questionable. A major driver of inconsistent data entry was the requirement to enter patient information simultaneously in both PBRs and Impilo, which participants described as unsustainable. They admitted that this dual documentation meant Impilo information was often not comprehensive.

*“If I need to make a report via Impilo, it does not give an accurate picture because some entries will be missing”,* KI_5

“*When a patient comes in from another department and their record was not updated online, I am forced to use a PBR instead. No official transfer to my department has been made for me to build upon. I have to retrieve information from the physical record or verbally from the escorting nurse.”* KI_3

*“I cannot trust information I find on Impilo. When I choose to use Impilo, I have to verify the data at the time because I constantly find information missing. This, to me, is a waste of time”.* KI_18

*What is the point of a digital system if the data is not complete? We might as well use paper.”* K1_14

#### Technical and infrastructural challenges.

The most pervasive barrier identified by participants was unreliable technical infrastructure, primarily frequent network downtime. HCWs reported that network interruptions caused disruption in data entry, and because Impilo did not offer an offline data entry option, work would stop completely during outages. Participants also noted that the existing solar power backup system was no longer efficient, and a more reliable backup power source was needed. The pharmacy department highlighted a specific consequence of this problem: prolonged logouts led to failure to update entries, resulting in inaccurate stock levels showing on the system. Furthermore, when the system was down for extended periods, staff often neglected to back-capture missed entries because this created extra workload.

“*At the pharmacy, if we stay for long logged off Impilo, we end up not updating previous entries. If entries are not updated, we have exaggerated stocks showing on the system”.* KI_4

“*If the system is down for long periods, other staff members ignore back capturing. Without backing up some information, it will not be found when needed. Back capturing creates extra workload for the HCWs, hence the tendency to ignore back capturing”.* KI_9

#### Human resource and workflow challenges.

HCWs, particularly nurses, expressed significant frustration with the requirement to write information in PBRs and simultaneously enter the same information into Impilo. They reported that this dual documentation often led them to abandon Impilo entirely, defaulting to PBRs to avoid increased workload. The chronic shortage of staff at the hospital exacerbated this problem, as HCWs faced competing demands for their time. Participants described having to choose between attending to waiting patients and completing data entry, with patient care invariably taking priority and data entry, particularly Impilo entry, being deferred and rarely completed later.

“*Imagine I have a number of patients all waiting to be attended to, then I take my time entering data in a register, and Impilo, I will simply not make progress with the patients at all. At the end of the day, I have to choose and I leave data entry-using Impilo, hoping I would later update. Honestly, I rarely update the information afterwards*”. KI_13

“*Using Impilo is tiresome for me; most of the burden of entering data from registers to Impilo is left to us to do, and this is not the only task I have during my working hours*”. KI_7

#### System design and inclusivity gaps.

A strong and distinct sub-theme emerged regarding gaps in system design and inclusivity. Participants reported that several departments, including X-Ray and Environmental Health, were not integrated into the Impilo ecosystem. Consequently, clinicians could not refer patients for X-ray or ultrasound examinations via Impilo, nor could radiology results be sent back through the system. Environmental Health Technicians responsible for notifiable disease surveillance could not access relevant information from their end because their department was simply not included. Laboratory staff noted that while they could enter results, the system did not automatically alert requesting clinicians, requiring manual checking that defeated the purpose of a digital system.

*“I cannot refer patients indicated for X-Ray or Ultrasound via Impilo because the feature is not there*”. KI_1

“*I have to do routine physical follow-up for updates on notifiable diseases because I cannot access such information from my end. My department is simply not included, hence nurses cannot refer cases using Impilo to the Environmental Health Department”.* KI_17

*“I can enter results, but the system doesn’t alert the requesting clinician. They have to manually check, which defeats the purpose,* KI_20.

### Suggestions to improve the utilisation of Impilo

#### Human resource & workflow changes.

HCWs strongly indicated a need for comprehensive training and technical support. Participants noted that, when training is limited to a few individuals, the remaining staff performed poorly and a lack of training for them fuelled underutilisation of the system. Even previously trained HCWs needed refresher sessions to keep up with system upgrades. Participants also described being stuck on certain features without anyone to assist them. A recurring suggestion was the introduction of dedicated data entry clerks to reduce the burden of dual documentation, allowing clinical staff to focus on patient care while clerks handled data entry.

“*Most of us nurse aides barely got any official training, but we are expected to be part of the people entering data on Impilo. We also need to be trained properly*”. KI_7

“*In my department, some HCWs will simply tell you they will not use something they were not trained for officially, hence the continued use of our paper registers only”.* KI_12

*“I failed to locate and make use of the inter-hospital transfer feature, and when I asked fellow colleagues, they were not aware of the feature”.* KI_10

*“We need dedicated data clerks. Let us do our nursing work, and let them handle the data entry. This would solve the dual documentation problem immediately.”* KI_19

#### Technical and infrastructural changes.

The majority of HCWs indicated that implementing offline data entry capability would significantly ease the burden of back-capturing. With offline functionality, they could continue entering data during network outages and simply upload when the system was back online, greatly improving consistency of data entry across departments. Participants also suggested that if offline functionality was not possible, investment in backup or alternative reliable internet sources would be beneficial.

*“If I consistently have access to Impilo at every point in time to enter data, I will end up getting used to it”.* KI_8

*“We have been using Impilo for over 2 years now, and we should have been relying on it only for data entry. With an option for offline data entry, I feel like we could have become accustomed to the system now and be fully operational. HCWs are demotivated by the frequent system interruptions”.* KI_10

#### Participatory system redesign.

HCWs highlighted a critical need for inclusive, participatory system redesign. They argued that involving diverse HCWs in the design process would facilitate the creation of more useful data fields and ensure all necessary departments were included. Participants noted that a user-friendly interface approved by HCWs themselves would improve adoption. Flexibility in data entry was also needed, as the narrow representation of patient histories on Impilo hindered HCWs from fully communicating client information. Some participants reported having to select the nearest diagnosis from a limited list, which misrepresented intended information.

“*I end up using the nearest diagnosis from the provided list, which misrepresents intended information*”. KI_9

“*The people who design Impilo should come and spend a week with us. They would see the real workflow and why some features don’t work. We are the users; our voice should be the loudest in the design process*.” KI_12

*“Why were we not consulted from the beginning? If they had asked, we would have told them the Environmental Health Department needs to be linked.”* (KI_17)

## Discussion

This qualitative study provides an in-depth exploration of the experiences of 20 healthcare workers with the Impilo EHR system in a rural Zimbabwean hospital. The findings illuminate a central paradox: HCWs recognise the significant potential benefits of Impilo for improving care but are systematically thwarted from realising them by a constellation of interdependent barriers. The research findings reveal that the system’s underutilisation stems not from resistance to technology itself, but from critical failures in the broader implementation ecosystem encompassing infrastructure, human resources, and organisational processes.

### Perceived benefits of Impilo

The findings indicate that HCWs perceive clear benefits of the Impilo system, primarily related to enhanced efficiency and data accessibility. The ease of retrieving patient information across departments and the reduction in medication errors due to the elimination of illegible handwriting were consistently highlighted. Our interpretation is that these benefits align closely with the core value proposition of EHRs globally, and HCWs in this rural setting are no less capable of recognising this value than their counterparts in better-resourced settings. This aligns with global evidence on EHRs, which positions them as crucial tools for improving continuity of care and patient safety [5,8]. Furthermore, features such as automated prompts and reminders were valued for enforcing data completeness, a finding consistent with studies that link such functionalities to improved guideline adherence [5]. The positive impact on inventory and resource management, especially within the pharmacy, highlights the potential for EHRs to extend beyond clinical care to strengthen health system supply chains, as supported by broader observations in similar contexts [18]. What is particularly striking is that despite extremely challenging working conditions, HCWs have not rejected the technology outright; rather, they express a desire for it to work properly a finding that speaks to their professionalism and commitment to improving patient care.

### Barriers to effective utilisation

The most pervasive barrier was the unreliable technical infrastructure, characterised by frequent network downtime and a lack of offline functionality. This finding is not merely descriptive but carries a critical implication for implementation policy: in rural low-resource settings, an “online-only” EHR system is fundamentally unsuitable [12,13]. We argue that offline-first architecture must be a non-negotiable design requirement for any digital health tool deployed in such environments. The direct consequence of this infrastructural weakness was the HCWs’ reliance on a dual documentation system, which created a significant increase in perceived workload and led to frustration and system abandonment. The direct consequence of this infrastructural weakness was the HCWs’ reliance on a dual documentation system, which created a significant increase in perceived workload and led to frustration and system abandonment. This represents a failure of the implementation strategy to account for the realities of the outer setting—the health system infrastructure and broader environment, a construct central to the Consolidated Framework for Implementation Research (CFIR) [27]

### System inclusivity and participatory design gaps

A crucial finding with major design implications was the significant gap in system inclusivity. The exclusion of key departments like Radiology and Environmental Health from the Impilo ecosystem created critical breaks in the digital patient journey, forcing HCWs to revert to paper-based and verbal communication. We interpret this finding as evidence of a top-down implementation approach that failed to engage end-users in the design process, a common failure in health IT implementation that has been well-documented in the literature [9,18]. The clear implication is that a participatory, co-design methodology must be institutionalised for all future development cycles, ensuring the system is built around the actual needs of all clinical and para-clinical departments. As one participant powerfully stated, “We are the users; our voice should be the loudest in the design process.” This sentiment echoes calls in the implementation science literature for user-centred design strategies [28,29].

### Actionable roadmap for intervention

The suggestions provided by HCWs offer a clear and actionable roadmap for intervention that directly addresses the identified barriers. The universal demand for offline functionality and the call for comprehensive, continuous training highlight critical gaps in the current implementation model. Our analysis suggests that sustainable adoption requires moving beyond one-off software deployment to a model of ongoing human capacity building and support, a factor consistently linked to success in similar African settings [15]. Furthermore, the proposal to employ dedicated data entry clerks is a strategic organisational intervention to resolve the workload paradox. We interpret this as a recognition by HCWs that the current expectation that already overburdened clinical staff should absorb additional data entry duties is fundamentally unrealistic. This carries a significant resource implication for policymakers: the cost of employing data clerks must be weighed against the cost of continued system failure and the lost productivity of clinical staff burdened with administrative tasks; a solution that has proven effective elsewhere [5]. The call for participatory system redesign is perhaps the most transformative suggestion, as it would address the root cause of many identified problems: the mismatch between system design and clinical reality.

### Comparison with other low-resource settings

The challenges identified in this study are not unique to Zimbabwe. Research from Tanzania has reported similar infrastructure and workload barriers to effective EHR utilisation in public primary healthcare facilities [18]. In Nigeria, inconsistent utilisation despite implementation across states has been attributed to inadequate training and support [15]. Recent studies from Ethiopia have similarly identified negligence in data recording during institutional births, with HCWs citing competing priorities and lack of supportive supervision as key barriers [16]. These parallels suggest that the barriers identified at St Anne’s are systemic across sub-Saharan Africa, reinforcing the need for regional guidance on EHR implementation in low-resource settings.

### Theoretical implication

This study makes several contributions to implementation science in low-resource settings. Applying the Consolidated Framework for Implementation Research (CFIR) [27], our findings demonstrate that the “outer setting” (infrastructure, connectivity, policy environment) is at least as important as the “inner setting” (organisational readiness, leadership engagement) when implementing digital health in rural Africa. Impilo’s underutilisation cannot be explained by individual HCWs’ resistance to change but rather by the failure to adapt the technology to the outer setting realities. Applying Normalization Process Theory (NPT) [30], the implementation failed because HCWs could not achieve “coherence” (understanding the system’s value when data was incomplete) or “cognitive participation” (committing to the system when it frequently failed). Future implementations should proactively assess these constructs before deployment.

### Strengths and limitations

This study has several strengths. The rigorous qualitative methodology, including thematic saturation, multi-coder analysis, member checking, and thick description, enhances the credibility and trustworthiness of the findings. The diversity of participants across professional roles and departments provides a comprehensive perspective on Impilo implementation. The study also provides rich contextual detail that supports transferability to similar settings.

Several limitations must be acknowledged. As a single-site study conducted at one rural mission hospital, the findings may not be fully generalisable to all health facilities in Zimbabwe, particularly urban or well-resourced facilities with more reliable infrastructure. The study is subject to potential social desirability bias, where participants may have under-reported negative experiences or over-reported positive ones despite assurances of confidentiality. However, the consistent and detailed accounts of challenges across multiple participants from different departments suggest that reporting was candid. Additionally, the study captured experiences at a single time point; longitudinal research would be valuable to understand how experiences evolve as the system and implementation strategies change. Finally, the study did not include non-user perspectives (e.g., patients, managers), which could provide additional insights into system impacts.

## Conclusion

The journey towards full digital transformation at St Anne’s Mission Hospital is stalled not by a rejection of technology, but by a failure to address the critical implementation barriers that make the technology a hindrance rather than a help. The barriers of unreliable infrastructure, inadequate training, and poorly adapted workflows are interlinked and must be tackled simultaneously. This study demonstrates that successful EHR implementation in resource-constrained settings requires a holistic strategy that goes beyond software installation to encompass robust infrastructure investment, sustained human capacity development, and genuine user engagement in system design

Based on our findings, we offer the following actionable recommendations:

For the Ministry of Health and Child Care and the Impilo technical team:

1. Prioritise development of offline-first functionality as a non-negotiable design requirement for all rural deployments.
2. Invest in redundant power solutions (e.g., upgraded solar systems with battery backup) at facilities with unreliable grid electricity.
3. Establish a continuous training and support programme, including refresher courses, on-site super-user support, and digital literacy enhancement for all HCWs.
4. Implement a formal participatory design process, including regular user feedback sessions and department-specific workflow analysis before system updates.

For hospital management at St Anne’s and similar facilities:

1. Employ dedicated data entry clerks to resolve the dual documentation burden, freeing clinical staff to focus on patient care.
2. Conduct a department-by-department needs assessment to identify missing features and integration requirements (e.g., Radiology, Environmental Health).
3. Establish clear protocols for back-capturing during network outages, with accountability mechanisms that do not unduly burden HCWs.
