# Supplementary material for: Perceived value but persistent barriers: A qualitative study of healthcare worker experiences with the Impilo electronic health record system in rural Zimbabwe
Source: PLOS Digit Health. 2026 Oct 8;5(10):e0001765. doi: 10.1371/journal.pdig.0001765 (PMC13648981; doi:10.1371/journal.pdig.0001765)
Supplement: Supplementary file 1 [file pdig.0001765.s001.docx]

**PEER REVIEW**

**Peer reviewer information:**

**Name:** AKINGENYE Josiane

**Location:** Kigali, Rwanda

**Job Title**: Director of Grants and Research

**Affiliation**: Informed Future Generation

**Date:** 13 April 2026

**Qualification**: PG in Clinical Research and Medication Writing, Associate degree in data science, and a Bachelor's in General Nursing.

**Manuscript title**: A Qualitative Study of Healthcare Worker Experiences with the Impilo Electronic Health Record System in Rural Zimbabwe

**Editor Summary-Minor Revisions Required**

The manuscript is well written, clear, and methodologically sound, with strong presentation of findings and discussion. The abstract and introduction are particularly well structured, and the study demonstrates good rigor overall.

Minor revisions are recommended to improve clarity and consistency, including:

- Specifying the time frame for the reported service population.
- Clarifying the distinction between the target population and the study sample.
- Ensuring consistency in describing participant characteristics (roles vs departments) and reflecting this in the demographic data.
- Standardizing participant coding throughout the manuscript.
- Providing additional detail on how coding discrepancies were resolved during data analysis.
- Strengthening the conclusion by including more explicit and actionable recommendations.

These are minor issues that, once addressed, will further enhance the clarity and quality of the manuscript.

**FEEDBACK**

1. **Abstract**

The abstract provides a clear and concise summary of the manuscript, with no jargon or technical terms, making it easy for non-medical readers to capture the message. It is informative, coherent, and logically structured, effectively capturing the key components of the study. Importantly, it successfully conveys the central message within the 257-word limit, which represents a notable strength.

2. **Introduction**

The introduction is clear and well-detailed, providing a comprehensive comparison of the global status of Electronic Health Records use across different regions, including Europe, the USA, and Africa, with specific examples from Tanzania (East Africa), Nigeria (West Africa), and Zimbabwe. This strengthens the study's contextual relevance.

3. **METHODS**
A. **Study Setting**

The site is clearly defined. However, while you state that the hospital serves approximately 3,073 people, the time frame is not specified (e.g., monthly or annually). Clarification is needed.

B. **Population and sampling**

The population and sampling are clearly described and relevant to the study design and objectives. Purposive sampling is appropriate, as it selects participants with relevant experience of the Impilo EHR system while ensuring diversity across departments, enabling the capture of varied experiences and challenges.

C. **Data Collection Tools and Procedure**

The data collection tool is well described, with its elements clearly aligned to the study objectives. Piloting the tool adds strength to the study. The data collection process is clear and follows appropriate scientific research procedures.

D. **Data analysis**

The data analysis process is well described and demonstrates rigor, including the use of multiple coders, iterative coding, and team-based theme refinement. These elements support the credibility of the findings. However, further clarification on how discrepancies between coders were resolved and whether additional strategies to enhance credibility were applied would strengthen this section.

4. **RESULTS, AND DISCUSSION**
A. **Demographic characteristics of participants**

- **There is a need for greater clarity and consistency in describing the study population. Earlier, the manuscript states that the target population included all 43 healthcare workers authorised to use the Impilo system across various departments. However, the results section reports that only 20 healthcare workers were interviewed. This distinction should be clearly explained to avoid confusion.**
- **Additionally, the manuscript refers to participants being drawn from different departments, yet the demographic description focuses mainly on the diversity of roles. Clarification is needed on whether departments were considered as participant characteristics. If so, they should be presented in the demographic table; if not, it would be more consistent to focus on roles throughout the manuscript.**
- **There is also an inconsistency in participant coding (e.g., “KI_1” versus “K1_3”), which should be standardized for clarity.**

B. **Results and Discussion**
   The results and discussion are well presented and effectively supported by comparisons with other studies, which strengthens the overall quality of the manuscript.
5. **Conclusion**

The conclusion is clear, coherent, and well-grounded in the study findings. However, it would be strengthened by including more explicit and actionable recommendations derived from the study findings.

6. **Summary of Minor Comments to Address**

- Clarify the time frame for the reported service population (3,073 people) in the study setting (e.g., monthly or annually).
- Provide additional detail on how discrepancies between coders were resolved during data analysis to strengthen credibility.
- Clearly distinguish between the target population (43 HCWs) and the actual sample (20 interviewed participants).
- Ensure consistency in describing participant characteristics (departments vs roles) and align this with the demographic table.
- Standardize participant coding format (e.g., “KI_1” vs “K1_3”) throughout the manuscript.
- Include more explicit and actionable recommendations in the conclusion.
