# Supplementary material for: Perceived value but persistent barriers: A qualitative study of healthcare worker experiences with the Impilo electronic health record system in rural Zimbabwe
Source: PLOS Digit Health. 2026 Oct 8;5(10):e0001765. doi: 10.1371/journal.pdig.0001765 (PMC13648981; doi:10.1371/journal.pdig.0001765)
Supplement: Supplementary file 2 [file pdig.0001765.s002.docx]

# Author's response to reviewers

#### MANUSCRIPT ID:   PDIG-D-25-00965

#### MANUSCRIPT TITLE: Perceived Value but Persistent Barriers: A Qualitative Study of Healthcare Worker Experiences with the Impilo Electronic Health Record System in Rural Zimbabwe.

| REVIEWER 1 COMMENT | AUTHOR RESPONSE | PAGE NUMBER |
| --- | --- | --- |
| General |  |  |
| Thank you for the opportunity to review the paper: “A Qualitative Study of Healthcare Worker Experiences with the Impilo Electronic Health Record System in Rural Zimbabwe” This is a well-written paper and he topic in this study is of high relevance related to the development of and use of EHR system in health services in the years to come. Use of qualitative method also gives the opportunity to dive into the field and get knowledge on how to develop further. | We thank the reviewer for the positive appraisal of our work and for recognizing the relevance of our study to the field of digital health in low-resource settings. |  |
| Introduction |  |  |
| 1. The introduction raises question on changes in the steering models and the values in developing a future of use of EHR in rural areas Zimbabwe. Globally there is challenges regarding how to document and share important health data related to for instance technology, organization, ethics, law, infrastructure, cultural context. This paper is a contribution to expand the knowledge and also the use of this kind of solutions. Several capacity building projects, e.g in Etiopia also focused on similar topics that may be of interest: Taye, B. K., Gezie, L. D., Atnafu, A., Mengiste, S. A., Kaasbøll, J., Gullslett, M. K., & Tilahun, B. (2025). Negligence in data recording during institutional birth: exploring health workers’ perspectives on the barriers and enablers of data documentation practices. Archives of Public Health, 83(1), 275. Ngusie, H. S., Kassie, S. Y., Zemariam, A. B., Walle, A. D., Enyew, E. B., Kasaye, M. D., ... & Mengiste, S. A. (2024). Understanding the predictors of health professionals' intention to use electronic health record system: extend and apply UTAUT3 model. BMC Health Services Research, 24(1), 889. | Thank you for these valuable suggestions. We have now cited both recommended studies in the Introduction to strengthen the contextual relevance and to highlight parallel findings from other African settings. The Taye et al. study on barriers to data recording during institutional birth is cited to support our discussion of dual documentation challenges. The Ngusie et al. study on UTAUT3 predictors of EHR intention to use is now cited to frame our findings around behavioural determinants of EHR use. | 7 |
| Theoretical approach |  |  |
| 2. Since this paper explores HCWs experiences with implementation of EHR Technology in health care services, the authors might concider to dive a bit into theories of innovation and implementation. This can be an opportunity to frame the findings and to develop and scale up use of HER across distance, organisations and professions.   Some suggested references:   - Damschroder, L. J., Reardon, C. M., Widerquist, M. A. O., & Lowery, J. (2022). The updated Consolidated Framework for Implementation Research based on user feedback. Implementation science, 17(1), 75.Nilsen, P. (2020). Making sense of implementation theories, models, and frameworks. In Implementation Science 3.0 (pp. 53-79). Cham: Springer International Publishing. - May, C., & Finch, T. (2009). Implementing, embedding, and integrating practices: an outline of normalization process theory. Sociology, 43(3), 535-554. - Powell, B. J., Waltz, T. J., Chinman, M. J., Damschroder, L. J., Smith, J. L., Matthieu, M. M., ... & Kirchner, J. E. (2015). A refined compilation of implementation strategies: results from the Expert Recommendations for Implementing Change (ERIC) project. Implementation science, 10(1), 21. - Proctor, E. K., Powell, B. J., & McMillen, J. C. (2013). Implementation strategies: recommendations for specifying and reporting. Implementation science, 8(1), 139. | This is an excellent suggestion. We have now integrated the Consolidated Framework for Implementation Research (CFIR) and Normalization Process Theory (NPT) into a new subsection in the Discussion titled "Theoretical Implications." This addition frames our findings within established implementation science frameworks, explaining how infrastructure failures (CFIR outer setting) and workflow integration challenges (NPT coherence and cognitive participation) explain Impilo's underutilisation. | 25 |
| Methods |  |  |
| 3. This section is well described related to the methods in use in this study, a descriptive qualitative study. Although it is well-written, the authors might consider exploring the epistemology/knowledge base of qualitative research, and position the knowledge-development in this paper in the field of qualitative research. Why is qualitative exploration important for this field and how can this knowledge contribute in this field and area?   Suggested references:   - Brinkmann, S. (2022). Qualitative interviewing: Conversational knowledge through research interviews. Oxford University Press. - Brinkmann, S., & Kvale, S. (2018). Doing interviews. https://www.torrossa.com/en/resources/an/5017966 - Golafshani, N. (2003). Understanding reliability and validity in qualitative research. The qualitative report, 8(4), 597-607. https://www.brown.uk.com/teaching/HEST5001/golafshani.pdf - Denzin, N. K., & Lincoln, Y. S. (Eds.). (2011). The Sage handbook of qualitative research. sage. | We thank the reviewer for this important methodological point. We have expanded the study design in the Methods section with the epistemological position of our study (interpretive/constructivist) and justifies why qualitative methods are essential for capturing lived experiences, contextual nuances, and the "why" behind implementation outcomes. Citations to Brinkmann (2022) and Golafshani (2003) have been added. | 9 |
| Discussion |  |  |
| 4. This section is also well written based on the presented material. If the author choose to include some of the suggested references, theoretical and methodologically, this can be included in the discussion as well. | As noted above, we have added a dedicated subsection in the Discussion that integrates CFIR and NPT. We have also referenced the methodological literature on qualitative rigour in the limitations section. | 22-25 |

| REVIEWER 2 COMMENT | AUTHOR RESPONSE | PAGE NUMBER |
| --- | --- | --- |
| General |  |  |
| 5. This is an interesting paper and the content is very good but I suggest the paper can be improved in the following ways | We thank the reviewer for the constructive feedback, which has significantly strengthened the manuscript. |  |
| Introduction |  |  |
| 6. Explain more the importance of study. You can talk about lack of qualitative studies and importance of them in explore experiences and … | We have expanded the Introduction to explicitly state the gap in qualitative literature on EHR experiences in rural Zimbabwe and to justify why qualitative exploration is necessary to understand the "lived experiences" of HCWs beyond quantitative utilisation metrics. | 7 |
| Methods |  |  |
| 7. Re-organize Methods section to this part: Design, Participants, Data collection, Data Analysis, Trustworthiness, and Ethical Considerations. | We have reorganised the Methods section exactly as requested, with clear subheadings. | 9-12 |
| 8. How did you select participants? Explain the process of purposeful sampling. theoretical sampling not correct for this study | We have corrected this. We now clearly describe purposive sampling, explain that we selected participants based on their authorisation to use Impilo, and detail how we ensured diversity across roles, departments, and experience levels. | 9-10 |
| 9. Add a reference to data saturation. | A reference to Guest et al. (2006) on data saturation in qualitative research has been added. | 10 |
| 10. Please provide questions and move to a table. | The semi-structured interview guide questions have been added as a new table (Table 1) in the Methods section. | 10-11 |
| 11. How did you design the questions? Explain it. | We have added a sentence explaining that questions were designed based on the study objectives (system usability, data quality, workflow changes, and suggestions for improvement) and informed by a literature review of EHR implementation challenges. | 10 |
| 12. Where did you do the interviews? Add the name of places. | We have specified that interviews were conducted in a private consultation room at St Anne's Mission Hospital. | 10 |
| 13. Before the start of each interview, did you talk about the study and it’s important to participant to get her/ his trust. If yes, mention it. | Yes. We have added a statement that before each interview, the researcher explained the study's purpose, assured confidentiality, and answered all questions to build rapport and trust. | 10 |
| 14. How did you protect the privacy of your participants? | We have added a detailed explanation: interviews were conducted in a private room, no identifying information was recorded, participant codes were used instead of names, and all data were stored on password-protected devices. | 10 |
| 15. Please indicate type of qualitative study | We have specified that this is a "descriptive qualitative study" (as already stated) and have now added a brief justification for this design choice. | 9 |
| Results |  |  |
| 16. Results are the main section of your research. It needs more describe in each category. Before quotations you have to describe the sub categories. 17. Emphasize that subcategories declares and bring quotations | We have substantially expanded the Results section. Before each quotation, we now provide descriptive narrative that explains the subcategory. Each subtheme is introduced with 2-3 descriptive sentences before the participant quotes are presented. | 15-21 |
| 18. Please mark the quotations in italic font | All participant quotations have been italicised throughout the Results section. | 15-21 |
| Discussion |  |  |
| 19. Discussion is well written, but it needs the interpretation of authors. Please add your interpretation and explanations to this section | We have added explicit interpretive statements throughout the Discussion. These include phrases such as "This finding suggests that...", "Our interpretation is that...", and "We argue that..." to clearly delineate author interpretation from descriptive findings. | 22-25 |
| 20. To strengthen the article, especially in the introduction and discussion section the following studies are suggested, please used and add to this manuscript references.  - The effect of bio ethical principles education on ethical attitude of prehospital paramedic personnel - -Iranian nurses' perceptions of core competencies required for disaster risk management - - Exploring the challenges of Iranian government hospitals related to Covid-19 pandemic management: qualitative content analysis research from the nurse’s perspective | We have carefully reviewed these suggested references. The articles are not directly focused on EHR implementation in low-resource settings |  |
| 21. Add limitations and strengthen of study. | We have expanded the Limitations section to include both limitations (single site, social desirability bias, potential transferability concerns) and explicitly stated strengths (rigorous qualitative methodology, thematic saturation, multi-coder analysis, thick description | 26 |
| 22. What are your suggestion for future studies? | Suggestions for future studies were incoperated on the Discussion section. | 22-25 |

| REVIEWER 3 COMMENT | AUTHOR RESPONSE | PAGE NUMBER |
| --- | --- | --- |
| General |  |  |
| 23. The manuscript is well written, clear, and methodologically sound, with strong presentation of findings and discussion. The abstract and introduction are particularly well structured, and the study demonstrates good rigor overall.   Minor revisions are recommended to improve clarity and consistency, including:   - Specifying the time frame for the reported service population. - Clarifying the distinction between the target population and the study sample. - Ensuring consistency in describing participant characteristics (roles vs departments) and reflecting this in the demographic data. - Standardizing participant coding throughout the manuscript. - Providing additional detail on how coding discrepancies were resolved during data analysis. - Strengthening the conclusion by including more explicit and actionable recommendations.   These are minor issues that, once addressed, will further enhance the clarity and quality of the manuscripts | We thank the reviewer for the positive assessment. All minor revisions have been addressed as detailed below. | Throughout the manuscript |
| Abstract |  |  |
| 24. The abstract provides a clear and concise summary of the manuscript, with no jargon or technical terms, making it easy for non-medical readers to capture the message. It is informative, coherent, and logically structured, effectively capturing the key components of the study. Importantly, it   successfully conveys the central message within the 257-word limit, which represents a notable strength. | No changes requested; we thank the reviewer. |  |
| Introduction |  |  |
| 25. The introduction is clear and well-detailed, providing a comprehensive comparison of the global status of Electronic Health Records use across different regions, including Europe, the USA, and Africa, with specific examples from Tanzania (East Africa), Nigeria (West Africa), and Zimbabwe. This strengthens the study & contextual relevance. | No changes requested; we thank the reviewer. |  |
| Methods |  |  |
| Study setting   26. The site is clearly defined. However, while you state that the hospital serves approximately 3,073 people, the time frame is not specified (e.g., monthly or annually). Clarification is needed. | We have clarified that the population of 3,073 people is from the 2022 Population and Housing Census and represents the total catchment population served annually. | 9 |
| Population and sampling   27. The population and sampling are clearly described and relevant to the study design and objectives. Purposive sampling is appropriate, as it selects participants with relevant experience of the Impilo EHR system while ensuring diversity across departments, enabling the capture of varied experiences and challenges. | No changes requested; we thank the reviewer. |  |
| Data Collection Tools and Procedure   28. The data collection tool is well described, with its elements clearly aligned to the study objectives. Piloting the tool adds strength to the study. The data collection process is clear and follows appropriate scientific research procedures. | No major changes. |  |
| Data analysis   29. The data analysis process is well described and demonstrates rigor, including the use of multiple coders, iterative coding, and team-based theme refinement. These elements support the credibility of the findings. However, further clarification on how discrepancies between coders were resolved and whether additional strategies to enhance credibility were applied would strengthen this section. | We have added detailed explanation: discrepancies were resolved through consensus meetings. This process is now explicitly described. | 11 |
| Results and Discussion |  |  |
| 30. The results and discussion are well presented and effectively supported by comparisons with other studies, which strengthens the overall quality of the manuscript. | Thank you. |  |
| Conclusion |  |  |
| 31. The conclusion is clear, coherent, and well-grounded in the study findings. However, it would be strengthened by including more explicit and actionable recommendations derived from the study findings. | We have substantially revised the Conclusion to include specific, actionable recommendations for the Ministry of Health, the Impilo technical team, and hospital management. These recommendations are now bulleted for clarity. |  |
| Summary of minor comments to address |  |  |
| 32. Clarify the time frame for the reported service population (3,073 people) in the study setting (e.g., monthly or annually). Provide additional detail on how discrepancies between coders were resolved during data analysis to strengthen credibility. Clearly distinguish between the target population (43 HCWs) and the actual sample (20 interviewed participants). | All addressed as noted above | 11 |
| 33. Ensure consistency in describing participant characteristics (departments vs roles) and align this with the demographic table. Standardize participant coding format (e.g., “KI_1” vs “K1_3”) throughout the manuscript. | We have standardised all participant codes to the format KI_1, KI_2, KI_3, etc., throughout the manuscript (previously there was an inconsistent "K1_14" format). The demographic table has been revised to ensure consistency between participant roles and the descriptions in the text. | Throughout the manuscript |
| 34. Include more explicit and actionable recommendations in the conclusion. | Done as noted above. | 27-28 |
