# Supplementary material for: Perceived value but persistent barriers: A qualitative study of healthcare worker experiences with the Impilo electronic health record system in rural Zimbabwe
Source: PLOS Digit Health. 2026 Oct 8;5(10):e0001765. doi: 10.1371/journal.pdig.0001765 (PMC13648981; doi:10.1371/journal.pdig.0001765)
Supplement: Peer review history

# Peer review: Decision Letter 0

**DOI:** 10.1371/journal.pdig.0001765.r001

**Authors:** Harry Hochheiser, Phat Kim Huynh

5 May 2026

Response to Reviewers Revised Manuscript with Track Changes Manuscript **Journal Requirements:**

Please confirm at this time whether or not your submission contains all raw data required to replicate the results of your study. Authors must share the “minimal data set” for their submission. PLOS defines the minimal data set to consist of the data required to replicate all study findings reported in the article, as well as related metadata and methods (https://journals.plos.org/plosone/s/data-availability#loc-minimal-data-set-definition).

For example, authors should submit the following data:

- The values behind the means, standard deviations and other measures reported;

- The values used to build graphs;

- The points extracted from images for analysis.

Authors do not need to submit their entire data set if only a portion of the data was used in the reported study.

If your submission does not contain these data, please either upload them as Supporting Information files or deposit them to a stable, public repository and provide us with the relevant URLs, DOIs, or accession numbers. For a list of recommended repositories, please see https://journals.plos.org/plosone/s/recommended-repositories.

**Additional Editor Comments (if provided):**

The primary reasons for rejection:

1. The study only looks at one rural hospital with 20 participants, making it hard to know if these findings apply elsewhere. The authors claim they reached "thematic saturation" but don't really explain how they determined this. Also, interviewing people two years after implementation means we're missing the early adoption challenges that might have been crucial.

2. While the authors mention using Braun and Clarke's thematic analysis, they don't provide enough detail about their actual coding process or inter-rater reliability. There's no theoretical framework guiding the study, which makes the findings feel somewhat disconnected from the broader implementation science literature.

3. The study only captures healthcare workers' views but ignores hospital management, IT staff, and patients. We also don't get any hard data - no usage statistics, completion rates, or actual performance metrics to back up what people are saying in interviews.

**Reviewers' Comments:**

**Comments to the Author**

1. Does this manuscript meet PLOS Digital Health’s publication criteria?

Reviewer #1: Yes

Reviewer #2: Yes

Reviewer #3: Yes

**********

2. Has the statistical analysis been performed appropriately and rigorously?

Reviewer #1: N/A

Reviewer #2: Yes

Reviewer #3: Yes

**********

3. Have the authors made all data underlying the findings in their manuscript fully available (please refer to the Data Availability Statement at the start of the manuscript PDF file)?

The PLOS Data policy

Reviewer #1: No

Reviewer #2: Yes

Reviewer #3: Yes

**********

4. Is the manuscript presented in an intelligible fashion and written in standard English?

Reviewer #1: Yes

Reviewer #2: Yes

Reviewer #3: Yes

**********

**Reviewer #1:**  PLOS review 01_2026

Dear editor and authors.

Thank you for the opportunity to review the paper: “A Qualitative Study of Healthcare Worker Experiences with the Impilo Electronic Health Record System in Rural Zimbabwe”

This is a well-written paper and he topic in this study is of high relevance related to the development of and use of of EHR system in health services in the years to come.

Use of qualitative method also gives the opportunity to dive into the field and get knowledge on how to develop further.

Introduction

The introduction raises question on changes in the steering models and the values in developing a future of use of EHR in rural areas Zimbabwe. Globally there is challenges regarding how to document and share important health data related to for instance technology, organization, ethics, law, infrastructure, cultural context. This paper is a contribution to expand the knowledge and also the use of this kind of solutions. Several capacity building projects, e.g in Etiopia also focused on similar topics that may be of interest:

Taye, B. K., Gezie, L. D., Atnafu, A., Mengiste, S. A., Kaasbøll, J., Gullslett, M. K., & Tilahun, B. (2025). Negligence in data recording during institutional birth: exploring health workers’ perspectives on the barriers and enablers of data documentation practices. Archives of Public Health, 83(1), 275.

Ngusie, H. S., Kassie, S. Y., Zemariam, A. B., Walle, A. D., Enyew, E. B., Kasaye, M. D., ... & Mengiste, S. A. (2024). Understanding the predictors of health professionals' intention to use electronic health record system: extend and apply UTAUT3 model. BMC Health Services Research, 24(1), 889.

Theoretical approach:

Since this paper explores HCWs experiences with implementation of EHR Technology in health care services, the authors might concider to dive a bit into theories of innovation and implementation. This can be an opportunity to frame the findings and to develop and scale up use of HER across distance, organisations and professions.

Some suggested references:

Damschroder, L. J., Reardon, C. M., Widerquist, M. A. O., & Lowery, J. (2022). The updated Consolidated Framework for Implementation Research based on user feedback. Implementation science, 17(1), 75.Nilsen, P. (2020). Making sense of implementation theories, models, and frameworks. In Implementation Science 3.0 (pp. 53-79). Cham: Springer International Publishing.

May, C., & Finch, T. (2009). Implementing, embedding, and integrating practices: an outline of normalization process theory. Sociology, 43(3), 535-554.

Powell, B. J., Waltz, T. J., Chinman, M. J., Damschroder, L. J., Smith, J. L., Matthieu, M. M., ... & Kirchner, J. E. (2015). A refined compilation of implementation strategies: results from the Expert Recommendations for Implementing Change (ERIC) project. Implementation science, 10(1), 21.

Proctor, E. K., Powell, B. J., & McMillen, J. C. (2013). Implementation strategies: recommendations for specifying and reporting. Implementation science, 8(1), 139.

Methods

This section is well described related to the methods in use in this study, a descriptive qualitative study. Although it is well-written, the authors might consider exploring the epistemology/knowledge base of qualitative research, and position the knowledge-development in this paper in the field of qualitative research. Why is qualitative exploration important for this field and how can this knowledge contribute in this field and area?

Suggested references:

Brinkmann, S. (2022). Qualitative interviewing: Conversational knowledge through research interviews. Oxford University Press.

Brinkmann, S., & Kvale, S. (2018). Doing interviews. https://www.torrossa.com/en/resources/an/5017966

Golafshani, N. (2003). Understanding reliability and validity in qualitative research. The qualitative report, 8(4), 597-607. https://www.brown.uk.com/teaching/HEST5001/golafshani.pdf

Denzin, N. K., & Lincoln, Y. S. (Eds.). (2011). The Sage handbook of qualitative research. sage.

Discussion

This section is also well written based on the presented material. If the author choose to include some of the suggested references, theoretical and methodologically, this can be included in the discussion as well.

**Reviewer #2:**  This is an interesting paper and the content is very good but I suggest the paper can be improved in the following ways:

Introduction

- Explain more the importance of study. You can talk about lack of qualitative studies and importance of them in explore experiences and …

Methods

- Re-organize Methods section to this part: Design, Participants, Data collection, Data Analysis, Trustworthiness, and Ethical Considerations.

- How did you select participants? Explain the process of purposeful sampling. theoretical sampling not correct for this study

- Add a reference to data saturation.

- please provide questions and move to a table.

- How did you design the questions? Explain it.

- Where did you do the interviews? Add the name of places.

Before the start of each interview, did you talk about the study and it’s important to participant to get her/ his trust. If yes, mention it.

- How did you protect the privacy of your participants?

-please indicate type of qualitative study

Results

- Results are the main section of your research. It needs more describe in each category. Before quotations you have to describe the sub categories.

-emphasize that subcategories declares and bring quotations

- Please mark the quotations in italic font

Discussion

- Discussion is well written, but it needs the interpretation of authors. Please add your interpretation and explanations to this section

- To strengthen the article, especially in the introduction and discussion section the following studies are suggested, please used and add to this manuscript references.

- The effect of bio ethical principles education on ethical attitude of prehospital paramedic personnel

-Iranian nurses' perceptions of core competencies required for disaster risk management

- Exploring the challenges of Iranian government hospitals related to Covid-19 pandemic management: a qualitative content analysis research from the nurses perspective

- Add limitations and strengthen of study.

- What are your suggestion for future studies?

**Reviewer #3:**  The manuscript is well written and methodologically sound, with clear presentation of findings. Minor revisions are needed to improve clarity and consistency, including participant description, coding consistency, data analysis details, and inclusion of more explicit recommendations in the conclusion.

**********

what does this mean?). If published, this will include your full peer review and any attached files.

**Do you want your identity to be public for this peer review?**  If you choose “no”, your identity will remain anonymous but your review may still be made public.

For information about this choice, including consent withdrawal, please see our Privacy Policy

Reviewer #1: No

Reviewer #2: No

Reviewer #3: **Yes:**  Josiane Akingeneye

**********

**Figure resubmission:**

**Reproducibility:**

To enhance the reproducibility of your results, we recommend that authors of applicable studies deposit laboratory protocols in protocols.io, where a protocol can be assigned its own identifier (DOI) such that it can be cited independently in the future. Additionally, PLOS ONE offers an option to publish peer-reviewed clinical study protocols. Read more information on sharing protocols at https://plos.org/protocols?utm_medium=editorial-email&utm_source=authorletters&utm_campaign=protocols

Attachment Submitted filename: Josiane A Peer Review-PDIG-D-25-00965.docx

# Author comment: Author response to Decision Letter 1

**DOI:** 10.1371/journal.pdig.0001765.r002

31 May 2026

Attachment Submitted filename: Reviewer Response.docx

# Peer review: Decision Letter 1

**DOI:** 10.1371/journal.pdig.0001765.r003

**Authors:** Harry Hochheiser, Phat Kim Huynh

20 Sep 2026

Perceived Value but Persistent Barriers: A Qualitative Study of Healthcare Worker Experiences with the Impilo Electronic Health Record System in Rural Zimbabwe.

PDIG-D-25-00965R1

Dear Mr Moyo,

We are pleased to inform you that your manuscript 'Perceived Value but Persistent Barriers: A Qualitative Study of Healthcare Worker Experiences with the Impilo Electronic Health Record System in Rural Zimbabwe.' has been provisionally accepted for publication in PLOS Digital Health.

Before your manuscript can be formally accepted you will need to complete some formatting changes, which you will receive in a follow-up email from a member of our team.

Please note that your manuscript will not be scheduled for publication until you have made the required changes, so a swift response is appreciated.

IMPORTANT: The editorial review process is now complete. PLOS will only permit corrections to spelling, formatting or significant scientific errors from this point onwards. Requests for major changes, or any which affect the scientific understanding of your work, will cause delays to the publication date of your manuscript.

If your institution or institutions have a press office, please notify them about your upcoming paper to help maximize its impact. If they'll be preparing press materials, please inform our press team as soon as possible -- no later than 48 hours after receiving the formal acceptance. Your manuscript will remain under strict press embargo until 2 pm Eastern Time on the date of publication. For more information, please contact digitalhealth@plos.org.

Thank you again for supporting Open Access publishing; we are looking forward to publishing your work in PLOS Digital Health.

Best regards,

Phat Kim Huynh, Ph.D.

Guest Editor

PLOS Digital Health

***********************************************************

**Additional Editor Comments (if provided):**

**Reviewer Comments (if any, and for reference):**

Reviewer's Responses to Questions

**Comments to the Author**

Reviewer #2: (No Response)

Reviewer #4: All comments have been addressed

Reviewer #5: All comments have been addressed

**********

publication criteria?

Reviewer #2: (No Response)

Reviewer #4: Yes

Reviewer #5: Yes

**********

3. Has the statistical analysis been performed appropriately and rigorously?

Reviewer #2: (No Response)

Reviewer #4: Yes

Reviewer #5: N/A

**********

4. Have the authors made all data underlying the findings in their manuscript fully available (please refer to the Data Availability Statement at the start of the manuscript PDF file)?

The PLOS Data policy

Reviewer #2: (No Response)

Reviewer #4: Yes

Reviewer #5: Yes

**********

5. Is the manuscript presented in an intelligible fashion and written in standard English?

Reviewer #2: (No Response)

Reviewer #4: Yes

Reviewer #5: Yes

**********

**Reviewer #2:**  Thank you

**Reviewer #4:**  The scientific content is strong: the study addresses a genuine gap in qualitative EHR-implementation research in rural Zimbabwe, sampling and saturation are well justified, the multi-coder thematic analysis and trustworthiness strategies (member checking, audit trail, thick description) meet accepted standards for rigor, and the new integration of CFIR/NPT substantially strengthens the Discussion. The reviewers' requests (interview guide as a table, standardized participant codes, italicized quotations, explicit author interpretation, actionable recommendations) have all been incorporated well in substance.

**Reviewer #5:**  Dear Editor,

Thank you very much for inviting me to review this manuscript. It has been a pleasure to contribute to the review process alongside the other reviewers, whose insightful and detailed comments I greatly appreciate.

The present manuscript entitled “Perceived Value but Persistent Barriers: A Qualitative Study of Healthcare Worker Experiences with the Impilo Electronic Health Record System in Rural Zimbabwe" addresses an important and impactful subject within its field of research.

Upon reviewing the initial and revised versions, the following points are evident:

• The authors have done a commendable job in addressing the reviewers’ comments thoroughly and constructively. They have logically incorporated all essential revisions to strengthen the manuscript. Notably, where certain requests appeared unjustified—such as suggestions to cite peripheral or unrelated studies—the authors appropriately and professionally refrained from doing so, maintaining the scholarly focus of the paper.

• Furthermore, integrating the foundational implementation and innovation theoretical frameworks recommended by Reviewer 1 (e.g., the updated Consolidated Framework for Implementation Research [CFIR], Normalization Process Theory [NPT], and implementation frameworks) has significantly enriched the conceptual depth, theoretical underpinning, and overall rigor of the manuscript.

• In light of these substantive improvements, I believe the revised version is in a very strong position and adequately addresses the peer-review feedback.

Considering the above-mentioned points and the authors' full compliance with the suggested revisions, the final version of the manuscript demonstrates sufficient scientific rigor and quality for publication. Accordingly, acceptance of the manuscript for publication in the journal is hereby confirmed.

We extend our appreciation to the authors for their valuable efforts in enhancing the quality of this research.

Yours sincerely,

**********

what does this mean?). If published, this will include your full peer review and any attached files.

**Do you want your identity to be public for this peer review?**  If you choose “no”, your identity will remain anonymous but your review may still be made public.

For information about this choice, including consent withdrawal, please see our Privacy Policy

Reviewer #2: None

Reviewer #4: No

Reviewer #5: None

**********
